# Supplementary material for: Impact of calculated plasma volume status on all-cause and cardiovascular mortality: 4-year nationwide community-based prospective cohort study
Source: PLoS One. 2020 Aug 20;15(8):e0237601. doi: 10.1371/journal.pone.0237601 (PMC7446862; doi:10.1371/journal.pone.0237601)
Supplement: Attachment — Submitted filename: Response to Reviewers.docx [file pone.0237601.s001.docx]

1. Is the manuscript technically sound, and do the data support the conclusions?

The manuscript must describe a technically sound piece of scientific research with data that supports the conclusions. Experiments must have been conducted rigorously, with appropriate controls, replication, and sample sizes. The conclusions must be drawn appropriately based on the data presented.

Reviewer #1: No

Reviewer #2: Yes

2. Has the statistical analysis been performed appropriately and rigorously?

Reviewer #1: Yes

Reviewer #2: Yes

3. Have the authors made all data underlying the findings in their manuscript fully available?

The [PLOS Data policy](http://www.plosone.org/static/policies.action#sharing) requires authors to make all data underlying the findings described in their manuscript fully available without restriction, with rare exception (please refer to the Data Availability Statement in the manuscript PDF file). The data should be provided as part of the manuscript or its supporting information, or deposited to a public repository. For example, in addition to summary statistics, the data points behind means, medians and variance measures should be available. If there are restrictions on publicly sharing data—e.g. participant privacy or use of data from a third party—those must be specified.

Reviewer #1: Yes

Reviewer #2: Yes

4. Is the manuscript presented in an intelligible fashion and written in standard English?

PLOS ONE does not copyedit accepted manuscripts, so the language in submitted articles must be clear, correct, and unambiguous. Any typographical or grammatical errors should be corrected at revision, so please note any specific errors here.

Reviewer #1: Yes

Reviewer #2: Yes

5. Review Comments to the Author
Please use the space provided to explain your answers to the questions above. You may also include additional comments for the author, including concerns about dual publication, research ethics, or publication ethics. (Please upload your review as an attachment if it exceeds 20,000 characters)

**Reviewer #1: The present study aimed to examine whether PVS is a novel risk factor for all-cause and cardiovascular deaths in the general population. It's an interesting study due to the variety of factors that could be related to cardiovascular mortality. However, the topic should be better explored.
Q1. The introduction section is really poor. It's not clear the relationship between plasma volume and mortality. What are the physiological mechanism that can explain this relationship ? Why PVS is associated with cardiac events and mortality in patients with heart failure ? Why is this discussion important and what are the hypothesis of the study?**
Answer to Q1. Thank you for your comments. We added the following sentences in Introduction section.

‘Heart failure remains a major and increasing public health problem, with a high mortality rate [4]. It was reported that imbalanced volume homeostasis causes systemic congestion and peripheral and pulmonary edema in heart failure [5]. Plasma volume expansion underlies systemic congestion, which is a well-known, clinically, and prognostically relevant complication of heart failure [6]. (Page 3, line 3)

‘American College of Cardiology/American Herat Association guidelines have recommended volume status be assessed [16]. (Page 3, line 15)’

‘Thus, we hypothesized that PVS may serve as an early identification of high-risk subjects for all-cause and cardiovascular deaths in the general population. (Page 3, line 18)’
**Q2. Methods. What changes is expected during the aging process in the PVS ? This answer can explain if the age range (40 to 74 years old) is too high (or not).**
Answer to Q2. Thank you for your question. To the best of our knowledge, there was no report regarding the relationship between aging and PVS. There was a negative correlation between aging and actual PV and ideal PV in the present study. On the other hand, there was a weak, but significant positive correlation between aging and PVS. These findings suggested that plasma volume may shift toward volume expansion with aging.

**Q3. In the “definition of PVS” section it is not clear where the values reported comes from (Men: a = 1530; b = 41; 3; c = 39 / women, a = 864; b = 47.9; c = 40). No reference is cited.**
Answer to Q3. Thank you for your suggestion. We added the citations.

**Q4 Is the authors used some statistical model to define the PVS range (7 and -13.3) ?
Is it possible a negative plasma volume ? It should be better explained.**

Answer to Q4. PVS is an index calculated by the following equation: (actual PV-ideal PV)/ideal PV, but not an estimated plasma volume itself. In TOPCAT study, PVS was reported to be less than 0 in 91% of heart failure patients (European Journal of Heart Failure. 2019; 21: 634-642).

Normal range of PVS has not been defined yet. In this study, we defined abnormally high and low PVS as PVS ≥ 7 and < -13.3, respectively, based on the results for 80% of all subjects.

We added the following sentences in Method section

‘Since aPV is often under iPV, the value of PVS could become less than 0. In the Treatment of Preserved Cardiac Function Heart Failure with an Aldosterone Antagonist Trial (TOPCAT) study, PVS was reported to be less than 0 in 91% of heart failure patients [13]. High and low PVS are considered as plasma volume expansion and contraction, respectively. (Page 7, line 8)’ **Q5. How blood pressure were measured ?**
Answer to Q5. Thank you for your question. We added the following sentence.

‘Blood pressure was measured the following method [17]. Participants were seated with back supports. After resting for at least 5 minutes, blood pressure was measured 2 times without conversation. Blood pressure was determined by an average of 2 blood pressure readings. (Page 6, line 12)

**Q6. Endpoint and follow-up section - All subjects were prospectively followed for a period of 914,292 person-years. (???????? Period should be in years/months/days). Participants were followed for 4 years. However the table one present the subject characteristics of the first year. How about the others ?**
Answer to Q6. As you pointed out, we rewrote the follow up period as follows. Since the purpose of the present study was to examine whether baseline PVS can predict clinical outcome in the general population, we showed the baseline characteristics in study subjects in Table 1.

‘All subjects were prospectively followed for a median follow up period of 4 years (interquartile range, 2.9-5.2 years; longest follow up, 7 years). (Page 8, line 1)
**Q7. The results were presented in a difficult way understand.
In the page 7 the authors comment: higher PVS - older and low risk / low PVS - younger and high risk. However, in page 8 and 9, high PVS was significantly associated with all-cause, cardiovascular and non-cardiovascular deaths / page 10 - low PVS was significantly associated with all-cause and cardiovascular deaths, but not non-cardiovascular deaths.
Which is better ? High or low ?**
Answer to Q7. High and low PVS mean imbalanced plasma volume in the general population. We considered the optimal PVS is associated with favor clinical outcome in the general population.

**Q8. The legend of figures are really poor. What does it means each line in the graphic ? The interpretation of the graphics are really difficult. The legends do not provide such informations.**
Answer to Q8. According to your comments, we modified figure legend.

‘We collected data from 230,989 subjects (aged 40–74 years) who participated in the health check-ups of 2008–2011. Among them, 107 were excluded from this study due to lack of essential data. During the median follow-up period of four years, there were 586 cardiovascular deaths, 2,552 non-cardiovascular deaths, and 3,138 all-cause deaths. (Page 20, line 2)’

‘Solid line shows the hazard ratios for subject groups stratified by 1% increments of PVS. Dotted lines show the 95% confidence interval for subjects stratified by 1% increments of PVS. Orange bar shows the distribution of study subjects. The referent group was defined as the lowest risk group. *P<0.05 v.s. referent group. (Page 20, line 7)

‘Green, red, and blue lines show the survival curves for high, normal, and low PVS groups, respectively. (Page 20, line 13)

**Q9. The first paragraph of discussion section shows a summary of the results. However the follow explanation it's not clean to a complete understanding of the relationship between PVS and cardiovascular risk.**
Answer to Q9. Thank you for your comments. We added the following sentences in Discussion section.

‘These findings indicated that imbalanced PVS may contribute to the development of cardiovascular disease. (Page 16, line 11)’

‘Potential explanation is that volume overload caused by plasma volume expansion may exacerbate cardiac function [6]. (Page 17, line 15)’

**Reviewer #2: The study now submitted is of good technical quality, its design was well prepared, presents extreme originality and offers great contributions to the application in the clinical area, including: early diagnosis, prognosis, emergency care and choices in making therapeutic decisions.**

**Q1. In addition, for a prospective epidemiological study, the Japanese territorial coverage was quite significant, Including more distant areas such as Okinawa, but no less important than the central areas.
However, on this point I have my first question: why did the researchers not include parts north of Honshu Island, in the representative Tohoku region, between Aomori and Fukoshima?**
Answer to Q1. Thank you for your question. Since we suffered from great earthquake in Tohoku region during study period, we could not obtain the follow-up data in this region unfortunately.

**Q2. The second issue concerns possible differences in PVS between higher altitude regions. As is well known, the plasma volume as well as the hematocrit may vary significantly between inhabitants of regions close to sea level and mountainous regions. This could interfere with the PVS values, the mean PVS obtained, over or underestimating mean values, as well as interfere with the inference of the analogies.
In the case of a Japanese archipelago with a wide variation in altitude, the authors do not point out such aspects, homogeneity or not of the altitude of the regions covered by the study and also what measures were taken to minimize possible errors.**
Answer to Q2. Thank you for your expertized question. We checked the residential area altitude. It ranged from sea level 31 m in Saitama to 644 m in Nagano. We found that there was a significant difference in PVS among region areas. Therefore, we added region area in multivariate analysis and calculation of C indices, NRI and IDI in order to adjust regional difference. PVS was still significantly associated with clinical outcomes and improved the prediction. We modified table 1, 2, and 3 and added the following sentences.
 ‘These prefectures were divided into four region areas; Hokkaido and Tohoku; Kanto and Koshinetsu; Kinki, Shikoku, and Chugoku; and Kyushu and Okinawa. (Page 6, line 5)

‘It was reported that high altitude affects volume status and its regulator hormone secretion [17], indicating the region difference in PVS. There was a significant difference in the prevalence of high and low PVS among region areas. (Page 11, line 20)’

**Q3. Another important question is, why were the mean PVS values of a random sample obtained from healthy people in the same age group and distributed in the regions of the study not obtained?**
Answer to Q3. Thank you for your comments. Since our study subjects were participants of health checkup, they were mainly apparently healthy subjects. Thus, we provide the data about PVS in healthy subjects.

**Q4. Finally, how do the authors justify the absence of the inclusion of the state of physical fitness or regular practice of physical activity or sedentary sedentary lifestyle among the variables analyzed and associated with PLWHA and its variations, since the regular practice of physical activity is recognized and widely used in the treatment of heart patients? Could its effects on the patient's clinical status and consequently on PLWHA not interfere in the discussion of the results?**

 Answer to Q4. Thank you for your suggestion. Unfortunately, we do not have the data about physical activity. Thus, we added the following sentence in Limitation section.

‘Finally, we do not have the data about physical activity, which may affect the future cardiovascular deaths. (Page 18, line 18)

6. PLOS authors have the option to publish the peer review history of their article ([what does this mean?](https://journals.plos.org/plosone/s/editorial-and-peer-review-process#loc-peer-review-history)). If published, this will include your full peer review and any attached files.


If you choose “no”, your identity will remain anonymous but your review may still be made public.


**Do you want your identity to be public for this peer review?** For information about this choice, including consent withdrawal, please see our [Privacy Policy](https://www.plos.org/privacy-policy).

Reviewer #1: No

Reviewer #2: Yes: Cassiano Merussi Neiva


[NOTE: If reviewer comments were submitted as an attachment file, they will be attached to this email and accessible via the submission site. Please log into your account, locate the manuscript record, and check for the action link "View Attachments". If this link does not appear, there are no attachment files to be viewed.]


While revising your submission, please upload your figure files to the Preflight Analysis and Conversion Engine (PACE) digital diagnostic tool, <https://pacev2.apexcovantage.com/>. PACE helps ensure that figures meet PLOS requirements. To use PACE, you must first register as a user. Registration is free. Then, login and navigate to the UPLOAD tab, where you will find detailed instructions on how to use the tool. If you encounter any issues or have any questions when using PACE, please email us at [figures@plos.org](mailto:figures@plos.org). Please note that Supporting Information files do not need this step.
